# Significance of fibulin-3 expression in bladder cancer: a tissue microarray-based immunohistochemical study

**DOI:** 10.1186/s12957-022-02597-z

**Published:** 2022-04-26

**Authors:** Ali Al Khader, Abdul Fattah S. Fararjeh, Ezidin G. Kaddumi, Mohamad Al-Saghbini

**Affiliations:** 1 Department of Pathology and Forensic Medicine, Faculty of Medicine, Al-Balqa Applied University, Al-Salt, Jordan; 2 Department of Medical Laboratory Analysis, Faculty of Science, Al-Balqa Applied University, Al-Salt, Jordan; 3 Department of Basic Medical Sciences, Faculty of Medicine, Al-Balqa Applied University, Al-Salt, Jordan; 4 Department of Pathology and Forensic Medicine, Faculty of Medicine, Al-Balqa Applied University, Al-Salt, Jordan

**Keywords:** Bladder, Cancer, Fibulin-3, Urothelial carcinoma

## Abstract

**Background:**

Predicting the behavior of bladder cancer by easy noninvasive methods and with less cost is needed. Fibulin-3 (EFEMP1), a glycoprotein of the extracellular matrix that is encoded by the gene *EFEMP1*, has been nominated as one of the potential mediators of muscle invasion in bladder cancer.

**Methods:**

In this tissue microarray-based immunohistochemical study, fibulin-3 level of expression was evaluated using a semiquantitative scoring system and was correlated with patient’s age and sex and tumor grade and stage.

**Results:**

A total of 160 urothelial carcinoma cases were analyzed. The age of the patients ranged from 25 to 91 years (mean, 60.15; SD, 11.60). Fibulin-3 was significantly associated with muscle invasion and overall tumor stage (*p* = 0.033 and 0.02, respectively). Fibulin-3 expression was nonsignificantly associated with tumor grade (*p* = 0.092)

**Conclusions:**

We found that the expression of fibulin-3 is significantly associated with muscle invasion in urinary bladder urothelial carcinoma. However, the prognostic role of fibulin-3 needs further investigations.

## Background

Urothelial carcinoma is the most common type of bladder cancer and is one of the most common cancers worldwide. It is classically classified into muscle invasive or muscle noninvasive and into low or high grade. In spite of the fact that most bladder cancers are of low grade and are non-muscle invasive, the risk of recurrence is high, and the financial burden of monitoring and treating bladder cancer patients is large. Moreover, the transformation into more aggressive clones occurs in about 10–20% of non-muscle-invasive bladder tumors [1]. Predicting the behavior of bladder cancer by easy noninvasive methods and with less cost is needed. Many urothelial carcinoma biomarkers have been recently discovered [2]. Fibulin-3 (EFEMP1), a glycoprotein of the extracellular matrix that is encoded by the gene *EFEMP1*, has been nominated as one of the potential mediators of muscle invasion in bladder cancer [3]. The role of fibulin-3 in different pathological processes including cancer has only been illustrated in the last 2 decades, and its function remains incompletely understood. It was firstly discovered as a protein that contains an EGF-like domain and that is encoded by the mRNA transcript S1-5 that was overexpressed in the fibroblasts in Werner syndrome, an early aging disease in human [4, 5]. Subsequent analysis of S1-5 at both mRNA and protein levels leads to its classification as one of the family of fibulins [6]. Human *EFEMP1* gene can be alternatively spliced to produce multiple fibulin-3 isoforms [4].

In this immunohistochemical study, we aim to analyze the expression of fibulin-3 in bladder cancer in relation to patient’s demographics and prognostic parameters represented by grade and presence or absence of muscle invasion.

## Methods

In this immunohistochemical study, we used tissue microarray (TMA) of human bladder cancer cases of different grades, stages, and patient characteristics. The TMA (serial no. BL2081b) was purchased from US Biomax. At US Biomax, the slides with whole tissue sections are viewed by a pathologist who also refers to the patient’s medical records to provide the pathology diagnosis. After the TMA block was constructed, the pathologist made an adjustment diagnosis by using the actual tissue cores staining results. The cases are graded according to the World Health Organization grading system [7]. The donor patients were from Asia.

We have performed immunohistochemical staining by rabbit monoclonal recombinant anti-fibulin-3 antibody (Abcam — serial no. ab356457, dilution 1:200) on these tissue sections. A semiquantitative method for scoring the staining was used by which the staining intensity (SI) was scored 0, 1, 2, and 3; if no staining, weak, moderate, or strong positivity was identified, respectively. The percentage of positivity (PP) was determined as 0, 1, 2, 3, or 4; if zero, < 10%, 10–50%, 50–75%, or > 75% positivity was identified, respectively. Histologic score of fibulin-3 (HS) was calculated as the product of PP and SI. The results of HS were interpreted as the following: 0 as negative, 1–5 as positive (low expression level), and 6–12 as positive (high expression level) [8]. The sections were blindly assessed by 2 experienced pathologists. Only bladder cancer cases of urothelial carcinoma type (*n* = 160) were chosen for analysis. HS was correlated with patient’s sex and age, in addition to tumor grade, muscle invasion, and overall stage. Statistical Package for the Social Sciences (SPSS) was used for analysis with Pearson chi-squared test being used and *p*-value < 0.05 being considered significant.

## Results

**Fig. 1:**
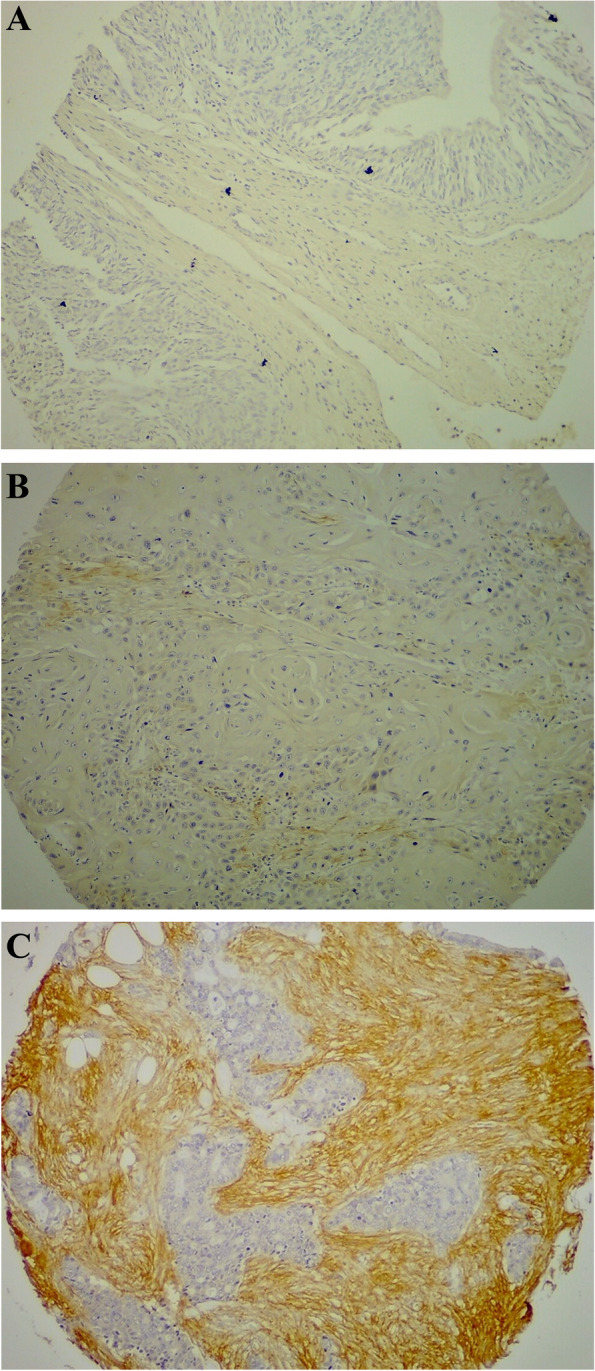
**A** Negative result of fibulin-3 immunostaining in a case of stage I urothelial carcinoma. **B** Low level of fibulin-3 expression in a case of stage II urothelial carcinoma. **C** High level of fibulin-3 expression in a case of stage III urothelial carcinoma

A total of 160 urothelial carcinoma cases were analyzed. The age of the patients ranged from 25 to 91 years (mean, 60.15; SD, 11.60). The age groups were determined as < 65, 65–84, and > 84 years. There was no significant association between patient’s age and level of fibulin-3 expression (*p* = 0.723). One-hundred twenty-five cases were of men and 25 were of women (M:F ratio 3.57:1) with no significant association between patient’s sex and fibulin-3 expression documented. The grade was known for 157 cases of which 15 were of grade I, 96 of grade II, and 46 of grade III. Fibulin-3 expression was nonsignificantly associated with tumor grade (*p* = 0.092). The overall stage was I, II, III, and IV for 50, 87, 21, and 2 cases, respectively. Fibulin-3 was significantly overexpressed in higher tumor stage compared to low stage (*p* = 0.02). Pathologic T stage was T1 (invasion of lamina propria), T2 (invasion of muscularis propria), and T3 (invasion of perivesical soft tissue) for 50, 87, and 23 cases, respectively. A significant association between fibulin-3 expression and pathologic T stage was found (*p* = 0.008). Fibulin-3 expression was also significantly associated with muscle invasion (*p* = 0.033). Table 1 shows the levels of fibulin-3 expression in relation to patient’s age, sex, tumor grade, stage, and muscle invasion status. Figure 1A, B, and C shows differential expression of fibulin-3 in a case of stage I, stage II, and stage III urothelial carcinomas, respectively.

**Table 1:** Differential expression of fibulin-3 in relation to patient’s age and sex and tumor grade and stage

Variable		No. of cases	Negative ***n***	Positive — low expression ***n***	Positive — high expression ***n***	***p***-value
Age (years)	< 65	93	36	23	34	0.723
	65–84	66	28	16	22	
	> 84	1	0	0	1	
Gender	Male	125	53	31	41	0.342
	Female	35	11	8	16	
Tumor grade	I	15	7	5	3	0.092
	II	96	44	22	30	
	III	46	12	11	23	
Tumor pathologic stage (pT)	pT1	50	22	17	11	0.008
	pT2	87	37	19	31	
	pT3	23	5	3	15	
Muscle invasion	Muscle invasive (pT2/T3)	110	42	22	46	0.033
	Muscle noninvasive (pT1)	50	22	17	11	
Overall stage	I	50	22	17	11	0.020
	II	87	37	19	31	
	III	21	5	3	13	
	IV	2	0	0	2

## Discussion

The secreted glycoprotein fibulin-3 has been known as a component of the extracellular matrix that is expressed in many normal adult and developing tissues. The ability for interaction with many other components of the extracellular matrix makes this glycoprotein implicated in different health as well as disease states [9]. The abnormal expression of fibulin-3 has been demonstrated in human cancer only recently [10]. In a study by Hu et al., they demonstrated that the overexpression of fibulin-3 in gliomas has a role in promoting tumor growth and invasion by acting as a soluble factor that activates Notch signaling through antagonizing the Notch autocrine inhibitor DLL3. They showed that the lockdown of the fibulin-3 gene *EFEMP1* increased apoptosis and impaired intracranial tumor growth, which was correlated with low expression levels of the genes related to Notch signaling in glioma samples [11]. Wang et al. applied plasmid transfection and added purified fibulin-3 in the cell lines of human osteosarcoma. They found that fibulin-3, through the induction by NF-κB signaling, mediated the crosstalk between AEG-1 and MMP-2 and was associated with poor prognosis of osteosarcoma due to invasion and metastases [12]. Moreover, in their recent study on the utility of serum fibulin-3 in diagnosing osteosarcoma and in predicting the prognosis, Wang et al. used ELISA in measuring the serum levels of fibulin-3 and found that serum fibulin-3 was significantly higher in osteosarcoma patients than in the control group and was significantly associated with higher stage and lung metastases [13]. In a study by Camaj et al., fibulin-3 was found to compete with EGF in binding EGFR and cause EGFR autophosphorylation in pancreatic adenocarcinoma with the subsequent signal transduction including Akt and MAPK phosphorylation, which may contribute to the tumor growth in pancreatic carcinomas with fibulin-3 overexpression. Furthermore, these findings pointed to the role of the inhibitor of EGFR kinase PD 153035 in inhibiting the downstream phosphorylation previously mentioned [14]. In Jiang et al.’s TMA-based study on different normal and mesothelioma tissues, fibulin-3 was found to be significantly expressed in mesothelioma in comparison with adjacent normal tissues, and this overexpression was correlated with poor survival in mesothelioma patients. In addition, that study showed that the overexpression of fibulin-3 was correlated with HMGB1 expression [15]. In leukemia, although promyelocytic leukemia is well known for its relatively good prognosis, Jann et al. analyzed whole transcriptomes of different acute myeloid leukemia cases and found that fibulin-3 was one of the most expressed factors in cases of early promyelocytic leukemia deaths in comparison with the cases with prolonged remission. Interestingly, EFEMP1 gene showed an almost exclusive expression in the early death cases [16]. In their immunohistochemical study on benign, borderline, and malignant ovarian tumors, Wang et al. showed that fibulin-3 was significantly associated with aggressive behavior of ovarian epithelial tumors. Moreover, they analyzed the relation of fibulin-3 expression with E-cadherin, N-cadherin, and snail expression in ovarian cancer. Interestingly, fibulin-3 overexpression was significantly associated with decreased E-cadherin expression, and increased N-cadherin and snail expression, which pointed to its role in epithelial-mesenchymal transition (EMT) [17]. However, fibulin-3 was found to increase E-cadherin expression and was associated with cell proliferation inhibition in endometrial carcinoma [18]. In Li et al.’s in vivo/in vitro study on the role of fibulin-3 in cervical cancer, fibulin-3 was significantly associated with malignant behavior and poor survival, and this was correlated with PI3K-Akt-mTOR pathway as well as EMT [19].

The role of *EFEMP1* as a tumor suppressor gene in breast cancer was illustrated in the study by Sadr-Nabavi et al. They found that *EFEMP1* was downregulated in a significant proportion of breast cancer cases as highlighted by the expression of RNA microarray and by real-time PCR. In addition, they revealed that this downregulation was attributed to epigenetic mechanisms through promoter methylation when the genomic sequencing on the cell lines and tissues of breast cancer was performed. Interestingly, their survival analysis of breast cancer cases revealed that reduced fibulin-3 expression was significantly associated with the overall and disease-free survivals in those who received anthracycline-based adjuvant chemotherapy for node-positive breast cancer [20]. The epigenetic mechanisms related to *EFEMP1* downregulation were also highlighted by Almeida et al. Through their microarray analysis of cell lines and tissue samples. They revealed that both histone deacetylation and promoter methylation contribute to the downregulation of *EFEMP1* that was significantly observed in prostate cancer in comparison with other urologic malignancies as well as normal prostatic tissues [21]. The role of *EFEMP1* promoter hypermethylation in cancer development was also documented in a study by Yue et al., in which a significant number of lung cancer tissues and cell lines showed *EFEMP1* promoter hypermethylation, while it was not found in the normal counterparts. *EFEMP1* promoter methylation was also suggested as an underlying mechanism for the low expression of fibulin-3 in hepatocellular carcinoma (HCC) [22]. In Hu et al.’s study on cell lines and tissues of HCC, *EFEMP1* was found to be significantly downregulated. They also demonstrated that its downregulation was associated with poor prognosis [23]. The downregulation of *EFEMP1* through epigenetic mechanisms was also reported in Yang et al.’s study on fibulin-3 in endometrial carcinoma. Their results suggested a role of fibulin-3 in promoting SEMA3B gene which has an important role in cell aging and death [18]. In Mao et al.’s study on fibulin-3 in colorectal cancer, *EFEMP1* was found to be downregulated, especially in the cell line SW480. Moreover, when SW480 cells were transfected with a fibulin-3 RNA-overexpressing lentivirus, tumor cell apoptosis was promoted, and cell proliferation was inhibited [24]. The underlying mechanisms for these findings remain ill-defined. However, in a recent study by Ying et al., fibulin-3 was positively correlated with TIMP1, and both were significantly associated with poor prognosis in rectal cancer [25].

The immunohistochemical staining results in this study showed that the overexpression of fibulin-3 was significantly associated with higher T and overall stage of bladder urothelial carcinoma. Although the role of fibulin-3 in promoting muscle invasion of bladder urothelial carcinoma is still poorly understood, the results of this study strongly supports the findings by Han et al. They used an integrative approach of RNA sequencing and using profiling studies of bladder cancer and found that fibulin-3 was significantly overexpressed in T2 tumors vs T1 ones. They confirmed fibulin-3 overexpression in muscle-invasive vs muscle noninvasive bladder cancer by quantitative reverse transcriptase PCR. Moreover, an interesting finding in that study was that fibulin-3 knockdown in a murine model of bladder cancer was associated with decreased expression of insulin-like growth factor-binding protein-5 (IGFBP5). This suggested that both fibulin-3 and IGFBP5 can be used as biomarkers for bladder cancer and are associated with an aggressive tumor behavior [3]. Many studies have been performed to uncover some of the complex molecular mechanisms of bladder cancer. Some of these studies pointed to possible diagnostic and prognostic markers. In a meta-analysis by Shi et al., microRNAs in the blood were shown to be useful diagnostic markers for urinary bladder cancer [26]. Another noninvasive promising biomarker is urine fibronectin. Dong et al. demonstrated that the combination of cytology with urine fibronectin can be essential for the detection of bladder cancer in clinical practice [27]. However, such findings need further validation using larger prospective studies. Bladder cancer shows significant molecular heterogeneity, and poor response to therapy represents a challenging event in bladder cancer tumorigenesis. The intratumoral heterogeneity displayed in bladder cancer is broad. In a recent study by Han et al., they revealed that low mutant-allele tumor heterogeneity may be a useful biomarker for better prognosis in *FGFR3*-mutant bladder cancer [28]. Several studies on the predictive role of certain biomarkers have been performed. For example, the long noncoding RNAs related to ferroptosis were shown to have a good predictive ability for overall survival and response to immunotherapy [29]. Regarding immunohistochemistry, similar to our study, many diagnostic and prognostic markers in bladder cancer have been studied. P53 overexpression, for example, was shown to be associated with progression of stage T1 bladder cancer [30]. Another example, the prolyl 3-hydroxylase family member 4 (P3H4), was associated with higher tumor grade and stage as highlighted in a recent study by Li et al. [31]. The abovementioned studies are just examples, and further investigations are still required.

## Conclusions

The results of this study support the limited data available on fibulin-3 expression in bladder cancer. We found that the expression of fibulin-3 is significantly associated with muscle invasion. However, further biomarker studies are still needed to confirm the prognostic role of fibulin-3 in bladder cancer, and the mechanisms underlying fibulin-3 role in aggressive bladder cancer behavior are still poorly understood.

## Data Availability

The datasets used during the current study are available from the corresponding author on reasonable request.

## Abbreviations

TMA: Tissue microarray
SI: Staining intensity
PP: Percentage of positivity
HS: Histologic score
SPSS: Statistical Package for the Social Sciences
EMT: Epithelial-mesenchymal transition
HCC: Hepatocellular carcinoma
P3H4: Prolyl 3-hydroxylase family member 4
